# Supplementary material for: Patterns of Intron Gain and Loss in Fungi
Source: PLoS Biol. 2004 Nov 30;2(12):e422. doi: 10.1371/journal.pbio.0020422 (PMC532390; doi:10.1371/journal.pbio.0020422)
Supplement: Table S1 — Also available at http://genes.mit.edu/NielsenEtAl/. (4.3 MB ZIP). [file pbio.0020422.st001.zip › NielsenEtAl/html/1358.html]

AN4397.1.NCU08935.1.MG06199.1.FG10362.1


```
 CLUSTAL W (1.82) Multiple Sequence Alignments - Introns Inserted


Sequence 1: NCU08935.1	531 aa
Sequence 2: MG06199.1	522 aa
Sequence 3: FG10362.1	514 aa
Sequence 4: AN4397.1	506 aa
Alignment Length: 537 aa
Number Identitical Residues: 294 aa
Alignment Score (without introns) 13452


MG06199.1 	MAAPNTLKGAIHGQ-PNATAVIIPTAKPGGAFLTVTYAGLVAEVSKFQAKLAAIGITQGS
NCU08935.1	MPCPFDLQAAIQGA-DNDVAVIIP-SKP--QALTVTYADLKRETASFQKKLADIGITKGS
FG10362.1 	---MSTLQSAINKSNPLAEAIIIP-SKP--KALTISYANLESDVSAFQRKLADLGITKAA
AN4397.1  	---MATLAQAFSPD-GSNAAVIVP-GKP---ALTVTYAQLHSHISAFQEKLAKLGVGHGA
          	      *  *:        *:*:* .**    **::** *  . : ** *** :*: :.:

MG06199.1 	AVSIATVNSYEFIVSFLAASWQRGIAAPLNPNYKQDEFEFYIEDVKSAIVLVPRGAFAAG
NCU08935.1	PVSIATVNSYEFIVSFLAASWQRGIAAPLNPAYKQDEFEFYIEDVKSAIVIVPKGAYAAG
FG10362.1 	PVSIALINSYEFIVSFLAASWQRGIAAPLNPAYKQDEFEFYIDDVKSAIVLVPRGAYAAG
AN4397.1  	AVNLALINSYEFIVGFLAASWQRAIAAPLNPAYKQDEFEFYIDDLSSTLVLIPRESYAQN
          	.*.:* :*******.********.******* **********:*:.*::*::*: ::* .

MG06199.1 	SPAVKAAKRFNAAVAECYWDAQKAEVALDVKDLGQLKGRGGQKVLEPQPEDTALVLHTS2
NCU08935.1	APVVKAAQKFNAAIAESYWDAAKNEVALDVKELGQLTGKGAQPVLKPDADDIALVLHTS~
FG10362.1 	APSVKAAKKFNAAVAECYWDEAKGEVALDVKDYGLLKGK-KERVLTAEPDDVALILHTS2
AN4397.1  	GPAVLAGRKYQAAIAECYWNG--TEVVLDVKELGKLKGKGDIGVQTAQPDDIALVLHTS~
          	.* * *.::::**:**.**:    **.****: * *.*:.   *  .:.:* **:**** 

MG06199.1 	GTTSRPKV0VPLSHRNLTRTMD1NIKNTYQLTPADRTMLVMPLFHVHGLLCGLLAPFYTG
NCU08935.1	GTTSRPKV~VPLTHRNLTRTMK~NIRNTYQLTSADRTMLVMPLFHVHGLLCGLLAPLLTG
FG10362.1 	GTTSRPKV0VPLTHRNLTRTMN1NIKNTYELTPKDRTMLVMPLFHVHGLLCGLLAPLFTG
AN4397.1  	GTTGRPKA0VPLTHKNLTTTMR1NIQATYKLTPQDRTYLVMPLFHVHGLLAAFLAPLASG
          	***.***. ***:*:*** **  **: **:**. *** ************..:***: :*

MG06199.1 	GSMVVPTKFSASDFWRDFVQHGANWYTAVPTIHQILLKNPAPSPLPKIRFIRSCSSPLSP
NCU08935.1	GSMIVPAKFSATDFWSDFIQFQANWYTAVPTIHQILLKHPVPNPLPKIRFIRSCSSPLSP
FG10362.1 	GSMVVPTKFSASEFWTDFNTHKANWYTAVPTIHQILLKNPPPSPKPNIRFIRSCSSPLSP
AN4397.1  	GSVIVPTKFSAHQFWSDFIEYKANWYSAVPTIHQILLKSPLPNPIPQIRFIRSCSSPLSP
          	**::**:**** :** **  . ****:*********** * *.* *:*************

MG06199.1 	TVFSQLEEKFGAPVLEAYAMTEAAHQMTSNPLP-PAKRKPGSVGIGQGVEVRILDGDGNE
NCU08935.1	TVFHNLEKTFQAPVLEAYAMTEAAHQMTSNPLPSVGKRKPGTVGLGQGVEVVILDDKDNI
FG10362.1 	TVFQQLEETYNAPVLEAYAMTEAAHQMTSNPLP-PAKRKAGTVGIGQGVEVKILTDQGEE
AN4397.1  	KTFQDLEKTLNAPVLEAYAMTEAAHQMTSNPLP-PAKRQPGSVGIGQGVEIKILDQSGNE
          	..* :**:.  **********************  .**:.*:**:*****: **  ..: 

MG06199.1 	LPRGVEGEICIRGENVTSGYLNNAEANASAFT-KGGFFRTGDQGKIDPEDGYVTITGRIK
NCU08935.1	LPDGQEGEISIRGENVTKGYLNNPEANASSFTRKGGYFRTGDQGKKD-EDGYIVITGRIK
FG10362.1 	VPQGTEGEICIIGENVTKGYLNNDKANAESYH-KNGFFRTGDQGKKD-EDGYIIITGRIK
AN4397.1  	VPQGHEAEICVRGENVTKGYLNNPAANKSSFT-KDGFFRTGDQGKKD-PDGYVIITGRIK
          	:* * *.**.: *****.*****  ** .::  *.*:******** *  ***: ******

MG06199.1 	ELINKAGEKISPIELDNVLTRHEAVSEAVSFAVPS-ELYGQDVGVAVVLKPG-AKLGKDE
NCU08935.1	ELINKGGEKISPIELDNVLTRHPAVSEAVSFAIPD-EMYGQEVAVAIVLKPGQQKVDAEE
FG10362.1 	ELINKGGEKISPIELDNVLTRHPQISEAVSFAIPD-EVYGQDIGVAVVLKNG-GKLTAQE
AN4397.1  	ELINKGGEKISPIELDNTLLQNPNVGEAVCFAIPDPGHYGEDIGAAVVLKSG-QNATEDE
          	*****.***********.* ::  :.***.**:*..  **:::..*:*** *  :   :*

MG06199.1 	LREWVAARLAKFKVPEK0IYYTE~IMPKTATGKIQRRIVAETMQKQDNKAK~L-------
NCU08935.1	FKQWVGSKLAKFKVPKK~IYFTD~VMPKTATGKIQRRIVAEEMLKKESRTT1GRVLRLTS
FG10362.1 	LKAWVEERSAKFKVPKK0VYFTE~VMPKTATGKIQRRIVAETMMTAP-KAK~L-------
AN4397.1  	LKSWVQEKLAKFKTPK-~----Q0QIPKTATGKIQRRKVAEAMLKPKAKL-~--------
          	:: **  : ****.*:      :  :*********** *** * .   :           

MG06199.1 	---
NCU08935.1	ARS
FG10362.1 	---
AN4397.1  	---
          	
```
